# Ancient DNA provides new insight into the maternal lineages and domestication of Chinese donkeys

**DOI:** 10.1186/s12862-014-0246-4

**Published:** 2014-11-30

**Authors:** Lu Han, Songbiao Zhu, Chao Ning, Dawei Cai, Kai Wang, Quanjia Chen, Songmei Hu, Junkai Yang, Jing Shao, Hong Zhu, Hui Zhou

**Affiliations:** Key-Lab for Evolution of Past Life and Environment in Northeast Asia, Jilin University, Ministry of Education, Changchun, 130012 PR China; Ancient DNA Laboratory, School of Life Sciences, Jilin University, Changchun, 130012 PR China; Research Center for Chinese Frontier Archaeology, Jilin University, Changchun, 130012 PR China; Archaeological Research Institute of Shaanxi Province, Xi’ an, 710054 PR China; Institute for Archaeology and Relic Conservation of Xi’ an, Xi’ an, 710068 PR China

**Keywords:** Chinese domestic donkeys, Ancient DNA, Mitochondrial DNA, D-loop, *Cytochrome b* gene, Maternal lineage, The Silk Road

## Abstract

**Background:**

The donkey (*Equus asinus*) is an important domestic animal that provides a reliable source of protein and method of transportation for many human populations. However, the process of domestication and the dispersal routes of the Chinese donkey are still unclear, as donkey remains are sparse in the archaeological record and often confused with horse remains. To explore the maternal origins and dispersal route of Chinese donkeys, both mitochondrial DNA D-loop and *cytochrome b* gene fragments of 21 suspected donkey remains from four archaeological sites in China were amplified and sequenced.

**Results:**

Molecular methods of species identification show that 17 specimens were donkeys and three samples had the maternal genetic signature of horses. One sample that dates to about 20,000 years before present failed to amplify. In this study, the phylogenetic analysis reveals that ancient Chinese donkeys have high mitochondrial DNA diversity and two distinct mitochondrial maternal lineages, known as the Somali and Nubian lineages. These results indicate that the maternal origin of Chinese domestic donkeys was probably related to the African wild ass, which includes the Nubian wild ass (*Equus africanus africanus*) and the Somali wild ass (*Equus africanus somaliensis*). Combined with historical records, the results of this study implied that domestic donkeys spread into west and north China before the emergence of the Han dynasty. The number of Chinese domestic donkeys had increased primarily to meet demand for the expansion of trade, and they were likely used as commodities or for shipping goods along the Silk Road during the Tang Dynasty, when the Silk Road reached its golden age.

**Conclusions:**

This study is the first to provide valuable ancient animal DNA evidence for early trade between African and Asian populations. The ancient DNA analysis of Chinese donkeys also sheds light on the dynamic process of the maternal origin, domestication, and dispersal route of ancient Chinese donkeys.

## Background

The process of domestication and dispersal route of the donkey (*Equus asinus*) is particularly interesting because the donkey has been widely used by humans to transport people and goods and till the land since approximately 5000 years ago [1-5]. The domestication of the donkey indicates a major cultural shift away from sedentary, agrarian life-styles towards more migration and trade [6].

China has the largest population in the world and a centuries-old history of raising donkeys [7]. In ancient times, the donkey played a critical role in agriculture and transportation, especially in arid and semiarid regions [3]. More recently, as farm mechanization and traffic automation have became more common, the number of donkeys has decreased sharply, though the ways they are used have greatly diversified [7,8]. Recently, there has been an increase in raising donkeys in China as donkey meat has been found to have high nutritional value and the Asini Corii Collas (donkey glue) is increasingly used as a component of traditional Chinese medicine [9,10]. Recent genetic research on the modern Chinese domestic donkey reveals that the modern Chinese donkey has an African maternal origin [11-13]. However, the domestication history and dispersal routes of the Chinese domestic donkey are still unclear.

In addition, it is challenging to distinguish donkey remains from horse remains using only morphological criteria [14]. First, the donkey and horse belong to the same genus *Equus*, which shares many similar morphological characters. Second, there are usually varying degrees of damage on ancient animal remains, which makes it difficult for morphological species identification. Molecular species identification offers a powerful tool to overcome the difficulties in species identification.

Mitochondrial DNA (mtDNA) has been widely used in species identification and genetic diversification of domestic animals because of its special characteristics such as: more copies than nuclear DNA, well-known gene structure, lack of introns, high mutation rate, and an absence of recombination events [15-17]. The mtDNA *cytochrome b* gene (*cytb*) is a very effective marker for species identification and phylogenetic studies because of its efficacy in revealing phylogenetic relationships in different families, genera, and species [10,18-20]. The mtDNA D-loop is a non-coding region and has a higher mutation rate than the *cytb* gene. For this reason, it has been extensively used to investigate the maternal origin and genetic diversification of domestic donkeys [21,22].

Beja-Pereira et al. [6] sampled domestic donkeys worldwide and sequenced both the mtDNA *cytb* gene and D-loop. Their phylogenetic results suggest that donkeys have an African maternal origin and exclude the possibility that the progenitors were Asiatic wild asses. Ten samples were from China, and the frequencies were 0.20 in the Nubian lineage and 0.80 in the Somali lineage [6]. Lei et al. [7] investigated the partial mtDNA D-loop sequences of 126 Chinese samples from 12 native breeds and supports that there is an African maternal origin for Chinese domestic donkeys [9]. Specifically, previous studies of Chinese domestic donkeys have focused on modern samples [6,9,20], which provide some information on the probable progenitor and gives insight into a possible dispersion model. However, these data cannot track the dynamic process of domestication in Chinese donkeys. It raises new questions about Chinese domestic donkeys. When were domestic donkeys introduced into China? What was the possible dispersal route for the ancient donkey to enter China?

Ancient DNA studies are needed to make up for this deficiency and to provide new insights into the domestication of livestock [23]. Kimura et al. [24] first analyzed ancient donkeys from archaeological sites and historic museums and found that the Nubian wild ass (*Equus africanus africanus*) was an ancestor of the first donkey haplogroup, but the Somali wild ass (*Equus africanus somaliensis*) was considerably diverged from the Nubian wild ass and domestic donkeys [24]. Until now, there have not been any genetic studies of ancient Chinese donkeys.

The goal of this study is to investigate the genetic structure of ancient Chinese donkeys and to explore the maternal origin and domestic history of Chinese domestic donkeys. We used ancient DNA methods to analyze 21 ancient samples from four Chinese archaeological sites. The partial mtDNA *cytb* gene and the D-loop sequences from ancient Chinese specimens were combined with previously published sequences for network and phylogenetic analysis. The results are used to better understand the maternal origins and dispersal routes of ancient Chinese donkeys, as well as the process of domestication.

## Results

### Species identification

It is a challenge to accurately identify species using morphology, especially when the animal remains have been damaged. Methods using molecular biology offer a powerful alternative to morphological methods for overcoming the difficulties in species identification. The *cytb* gene, in particular, can be used in DNA barcoding for species identification. In this study, most of the ancient samples were identified as donkeys using mtDNA *cytb* gene analysis, and these results are consistent with species identification through traditional morphological methods. However, three samples (L7, L8 and L11) had the maternal genetic signature for horses using both the *cytb* gene and the D-loop sequences. These samples came from only one bone or tooth and had been provisionally identified as donkeys by morphological methods (Table 1). The three samples were probably horses or mules, which are the offspring of a cross between a male donkey and a female horse. In actuality, mule remains are difficult to detect archaeologically, because their bones or teeth cannot be reliably distinguished from horses and donkeys [25]. These results show the power of methods using molecular biology in species identification of ancient samples.

**Table 1:** **Archaeological samples studied, with associated codes, elements used, dates, and results**

**Lab code**	**Archaeological site**	**Archaeological code**	**Element**	**Date (yr BP)**	**Result** ^*^
L1	Yanjialiang	06BJYT2125③:12	bone	800-600	Donkey
L2	Yanjialiang	06BJY:6934	bone	800-600	Donkey
L3	Yanjialiang	06BJY6866	bone	800-600	Donkey
L4	Yanjialiang	06BJY:6419	bone	800-600	Donkey
L5	Yanjialiang	06BJY1135④:62	bone	800-600	Donkey
L6	Yanjialiang	06BJY1332②:54	bone	800-600	Donkey
L7	Yanjialiang	06BJY1231H78:8	bone	800-600	Horse
L8	Yanjialiang	06BJYH64:20	bone	800-600	Horse
L9	Yanjialiang	06BJY:5116	bone	800-600	Donkey
L10	Jinsitai cave	01DAJT3④:3	tooth	20,000-18,000	Failure
L11	Yanjialiang	06BJY:788	tooth	800-600	Horse
L12	Yanjialiang	06BJYT1132④:23	tooth	800-600	Donkey
L13	Yanjialiang	06BJY:2666	bone	800-600	Donkey
L14	Yanjialiang	06BJY:7474	bone	800-600	Donkey
L15	Yanjialiang	06BJY:7575	bone	800-600	Donkey
L16	Yanjialiang	06BJY:7676	bone	800-600	Donkey
L17	Yanjialiang	06BJYT1135④:119	bone	800-600	Donkey
L18	Yanjialiang	06BJY:803	bone	800-600	Donkey
L19	Xi'an Wanke	M1-D1:0219	tooth	1,160±30	Donkey
L20	Xi'an Wanke	M1-D1:0220	tooth	1,160±30	Donkey
L21	Lantianxinjie	09K1-F3:1	bone	568±50	Donkey

^*^Results of species identification by using both the mtDNA *cytb* gene and D-loop sequences.

### MtDNA variation and haplotypes

We successfully acquired 20 mtDNA *cytb* gene sequences of 448 bp using two pairs of primers 7F/7R and 8F/8R (Table 2). The final size of analyzed sequences was 366 bp. The data in this paper have been deposited into GenBank with accession numbers: KM235000-KM235019. The sequences for the *cytb* gene revealed that there were 4 different haplotypes with 36 polymorphic sites. One unique haplotype and 3 shared haplotypes were found among these samples. There were no insertions or deletions observed in the 20 mtDNA *cytb* gene sequences. 33 transitions and 3 transversions (15066 T/A, 15105 A/C and 15204 C/A) were identified, suggesting a strong bias towards transitions. 29 polymorphic sites were used to distinguish between donkey and horse remains (Table 3). The results revealed that both pairs of primers 7F/7R and 8F/8R are species-specific primers and can distinguish between donkeys and horses in this study.

**Table 2:** **Primers and annealing temperatures for PCR amplification**

**MtDNA**	**Primers**		**Sequence(5’-3’)**	**Annealing temperature**	**Length**
D-loop	1F/1R	L15424	CACCATCAACACCCAAAGCT	50.8°C	201 bp
		H15625	ACATGCTTATTATTCATGGGGC		
	6F/6R	L15599	GCCCCATGAATAATAAGCA	51.5°C	257 bp
		H15855	TGAAGAAAGAACCAGATGCC		
	4F/5R	L15828	TGAAACTATACCTGGCATCTGG	51.5°C	280 bp
		H16107	CATGGACTGAATAACACCTTATGG		
*Cytb*	7F/7R	L14936	GAGACCCAGACAACTACACC	50.5°C	235 bp
		H15170	ACTAAGAGTCAGAACACGCA		
	8F/8R	L15133	TTCCGACCCCTTAGTCAATG	52.6°C	251 bp
		H15383	AATGTTTCCCCCTTTTCTGG		

The positions for the complete mitochondrial genome are at GenBank X97337.

**Table 3:** **Distribution of 36 observed polymorphic sites in the mtDNA** ***cytb*** **gene sequences of Chinese ancient samples**

	**1**	**1**	**1**	**1**	**1**	**1**	**1**	**1**	**1**	**1**	**1**	**1**	**1**	**1**	**1**	**1**	**1**	**1**	**1**	**1**	**1**	**1**	**1**	**1**	**1**	**1**	**1**	**1**	**1**	**1**	**1**	**1**	**1**	**1**	**1**	**1**	**Species/Lineage**
	**4**	**5**	**5**	**5**	**5**	**5**	**5**	**5**	**5**	**5**	**5**	**5**	**5**	**5**	**5**	**5**	**5**	**5**	**5**	**5**	**5**	**5**	**5**	**5**	**5**	**5**	**5**	**5**	**5**	**5**	**5**	**5**	**5**	**5**	**5**	**5**	
	**9**	**0**	**0**	**0**	**0**	**0**	**0**	**0**	**0**	**0**	**0**	**1**	**1**	**1**	**1**	**1**	**1**	**1**	**1**	**1**	**1**	**2**	**2**	**2**	**2**	**2**	**2**	**2**	**2**	**2**	**2**	**2**	**3**	**3**	**3**	**3**	
	**9**	**0**	**0**	**1**	**5**	**5**	**6**	**7**	**7**	**8**	**9**	**0**	**1**	**3**	**3**	**4**	**4**	**4**	**8**	**9**	**9**	**0**	**0**	**1**	**1**	**3**	**4**	**7**	**7**	**7**	**9**	**9**	**0**	**1**	**2**	**5**	
	**1**	**3**	**9**	**5**	**1**	**4**	**6**	**2**	**9**	**1**	**9**	**5**	**1**	**2**	**8**	**1**	**4**	**7**	**7**	**8**	**9**	**1**	**4**	**1**	**3**	**1**	**3**	**0**	**6**	**7**	**4**	**6**	**1**	**8**	**3**	**5**	
X97337	G	T	A	T	T	T	T	T	T	A	T	A	G	G	A	C	T	T	C	T	G	T	C	G	A	C	G	T	C	T	C	C	G	G	G	T	Donkey/Somali
L1	.	.	.	.	.	.	.	.	.	.	C	.	.	.	.	.	.	.	T	C	.	.	.	.	.	.	.	.	.	.	.	.	.	A	.	.	Donkey/Somali
L5	.	.	.	.	.	.	.	.	.	.	C	.	.	.	.	.	.	.	T	C	.	.	.	.	.	.	.	.	.	.	.	.	.	A	.	.	Donkey/Somali
L14	.	.	.	.	.	.	.	.	.	.	C	.	.	.	.	.	.	.	T	C	.	.	.	.	.	.	.	.	.	.	.	.	.	A	.	.	Donkey/Somali
L15	.	.	.	.	.	.	.	.	.	.	C	.	.	.	.	.	.	.	T	C	.	.	.	.	.	.	.	.	.	.	.	.	.	A	.	.	Donkey/Somali
L16	.	.	.	.	.	.	.	.	.	.	C	.	.	.	.	.	.	.	T	C	.	.	.	.	.	.	.	.	.	.	.	.	.	A	.	.	Donkey/Somali
L18	.	.	.	.	.	.	.	.	.	.	C	.	.	.	.	.	.	.	T	C	.	.	.	.	.	.	.	.	.	.	.	.	.	A	.	.	Donkey/Somali
L19	.	.	.	.	.	.	.	.	.	.	C	.	.	.	.	.	.	.	T	C	.	.	.	.	.	.	.	.	.	.	.	.	.	A	.	.	Donkey/Somali
L2	.	.	.	.	.	.	.	.	.	.	.	.	.	.	.	.	.	.	.	.	.	.	.	.	.	.	.	.	.	.	.	.	.	.	.	.	Donkey/Nubian
L3	.	.	.	.	.	.	.	.	.	.	.	.	.	.	.	.	.	.	.	.	.	.	.	.	.	.	.	.	.	.	.	.	.	.	.	.	Donkey/Nubian
L4	.	.	.	.	.	.	.	.	.	.	.	.	.	.	.	.	.	.	.	.	.	.	.	.	.	.	.	.	.	.	.	.	.	.	.	.	Donkey/Nubian
L6	.	.	.	.	.	.	.	.	.	.	.	.	.	.	.	.	.	.	.	.	.	.	.	.	.	.	.	.	.	.	.	.	.	.	.	.	Donkey/Nubian
L9	.	.	.	.	.	.	.	.	.	.	.	.	.	.	.	.	.	.	.	.	.	.	.	.	.	.	.	.	.	.	.	.	.	.	.	.	Donkey/Nubian
L13	.	.	.	.	.	.	.	.	.	.	.	.	.	.	.	.	.	.	.	.	.	.	.	.	.	.	.	.	.	.	.	.	.	.	.	.	Donkey/Nubian
L17	.	.	.	.	.	.	.	.	.	.	.	.	.	.	.	.	.	.	.	.	.	.	.	.	.	.	.	.	.	.	.	.	.	.	.	.	Donkey/Nubian
L20	.	.	.	.	.	.	.	.	.	.	.	.	.	.	.	.	.	.	.	.	.	.	.	.	.	.	.	.	.	.	.	.	.	.	.	.	Donkey/Nubian
L21	.	.	.	.	.	.	.	.	.	.	.	.	.	.	.	.	.	.	.	.	.	.	.	.	.	.	.	.	.	.	.	.	.	.	.	.	Donkey/Nubian
L12	.	.	.	.	.	.	.	.	.	.	.	.	.	.	.	.	.	.	.	.	A	.	.	A	.	.	.	.	.	.	.	.	.	.	A	.	Donkey/Nubian
L7	A	C	G	C	C	C	A	C	C	G	C	C	A	A	G	T	C	C	.	.	.	C	A	.	G	T	A	C	T	C	T	T	A	A	.	C	Horse
L8	A	C	G	C	C	C	A	C	C	G	C	C	A	A	G	T	C	C	.	.	.	C	A	.	G	T	A	C	T	C	T	T	A	A	.	C	Horse
L11	A	C	G	C	C	C	A	C	C	G	C	C	A	A	G	T	C	C	.	.	.	C	A	.	G	T	A	C	T	C	T	T	A	A	.	C	Horse

X97337 as the reference sequence from GenBank.

We successfully acquired 20 mtDNA D-loop sequences of 684 bp using three pairs of primers: 1F/1R, 6F/6R, and 4F/5R (Table 2). Final analyzed sequences range in size from 577-578 bps. All D-loop sequences are available through GenBank (KM234980- KM234999). Three of them belong to horses (KM234980-KM234982). In this study, we focused on the phylogenetic relationship between 17 mtDNA D-loop sequences of donkeys (KM234983-KM234999). These sequences showed that there were ten different haplotypes with 28 polymorphic sites (Table 4). There were 26 transitions, one transversion (15653 A/C), and one deletion (15863 deletion), suggesting a strong bias towards transitions. Of the ten different haplotypes, there were six unique haplotypes and four shared haplotypes among the ancient Chinese donkeys. The most frequent haplotype was H7, which was found in three samples (L1, L5 and L14) from the Yanjialiang site and one sample (L19) from the Xi'an Wanke site. The haplotype diversity (Hd) and nucleotide diversity (π) for the ancient Chinese donkeys are 0.919 and 0.01815, respectively, suggesting that there was high genetic diversity in the ancient Chinese donkey population.

**Table 4:** **Polymorphic sites for the mtDNA D-loop haplotypes in ancient Chinese donkeys**

**Haplotypes**	**Nucleotide positions**																												**Samples**	**Lineage**
	1	1	1	1	1	1	1	1	1	1	1	1	1	1	1	1	1	1	1	1	1	1	1	1	1	1	1	1		
	5	5	5	5	5	5	5	5	5	5	5	5	5	5	5	5	5	5	5	5	5	5	5	5	5	5	5	5		
	4	4	5	5	5	5	5	5	6	6	6	6	6	6	6	6	7	7	7	8	8	8	8	8	8	9	9	9		
	8	9	0	3	6	8	9	9	4	4	4	5	5	6	9	9	0	6	7	0	0	2	2	2	6	5	9	9		
	4	0	3	1	9	0	8	9	4	5	9	2	3	2	5	8	1	4	0	1	6	0	1	2	3	1	0	1		
X97337	G	C	T	C	A	A	C	A	G	A	C	C	A	A	C	C	A	C	T	C	C	C	G	G	T	G	G	T		
H1	.	.	.	.	.	.	.	G	.	.	.	.	.	.	.	.	.	.	.	.	.	.	.	.	-	.	A	.	L2,L3,L4	Nubian
H2	.	.	.	.	.	G	.	G	.	.	.	.	.	.	.	.	G	.	.	.	.	.	.	.	-	.	A	.	L17	Nubian
H3	.	.	.	.	.	.	.	G	.	.	.	.	.	.	.	.	.	.	.	.	.	.	A	.	-	.	A	.	L9,L20	Nubian
H4	.	.	.	.	.	.	.	G	A	.	T	.	C	.	T	.	.	T	.	.	.	.	.	.	-	.	A	.	L12	Nubian
H5	.	.	.	.	.	.	.	G	A	.	.	.	.	.	T	.	.	.	.	.	.	.	.	.	-	.	A	.	L13,L21	Nubian
H6	.	.	.	.	G	.	.	G	.	.	.	.	.	.	T	.	.	.	.	.	.	.	.	.	-	.	A	.	L6	Nubian
H7	A	T	C	.	G	G	T	G	A	.	.	T	.	G	.	T	.	.	C	T	T	T	A	A	-	A	A	C	L1,L5,L14,L19	Somali
H8	A	T	C	T	G	G	T	G	A	.	.	T	.	G	.	T	.	.	C	T	T	T	A	A	-	A	A	C	L15	Somali
H9	A	T	C	.	G	G	T	G	A	G	.	T	.	G	.	T	.	.	C	T	T	T	A	A	-	A	A	C	L16	Somali
H10	A	T	C	.	G	G	T	G	A	G	.	T	.	G	.	T	.	.	C	T	.	T	A	A	-	A	A	C	L18	Somali

X97337 indicates the nucleotides for 15484-15991, according to the GenBank accession number X97337.

The PCR from the sample L10 that came from the Jinsitai cave site failed to amplify. Some potential causes include: (i) L10 is from 20,000-18,000 yr BP and was too old for successful ancient DNA amplification and (ii) L10 was treated with white emulsion for sample protection, which may have reduced the DNA content and damaged the ancient DNA.

The mtDNA D-loop has a higher mutation rate than the *cytb* gene. Thus, the D-loop is more suitable for phylogenetic study within species, while the *cytb* gene is mainly used for species identification and lineage divergence.

### Phylogenetic tree and reduced median network construction

**Figure 1:**
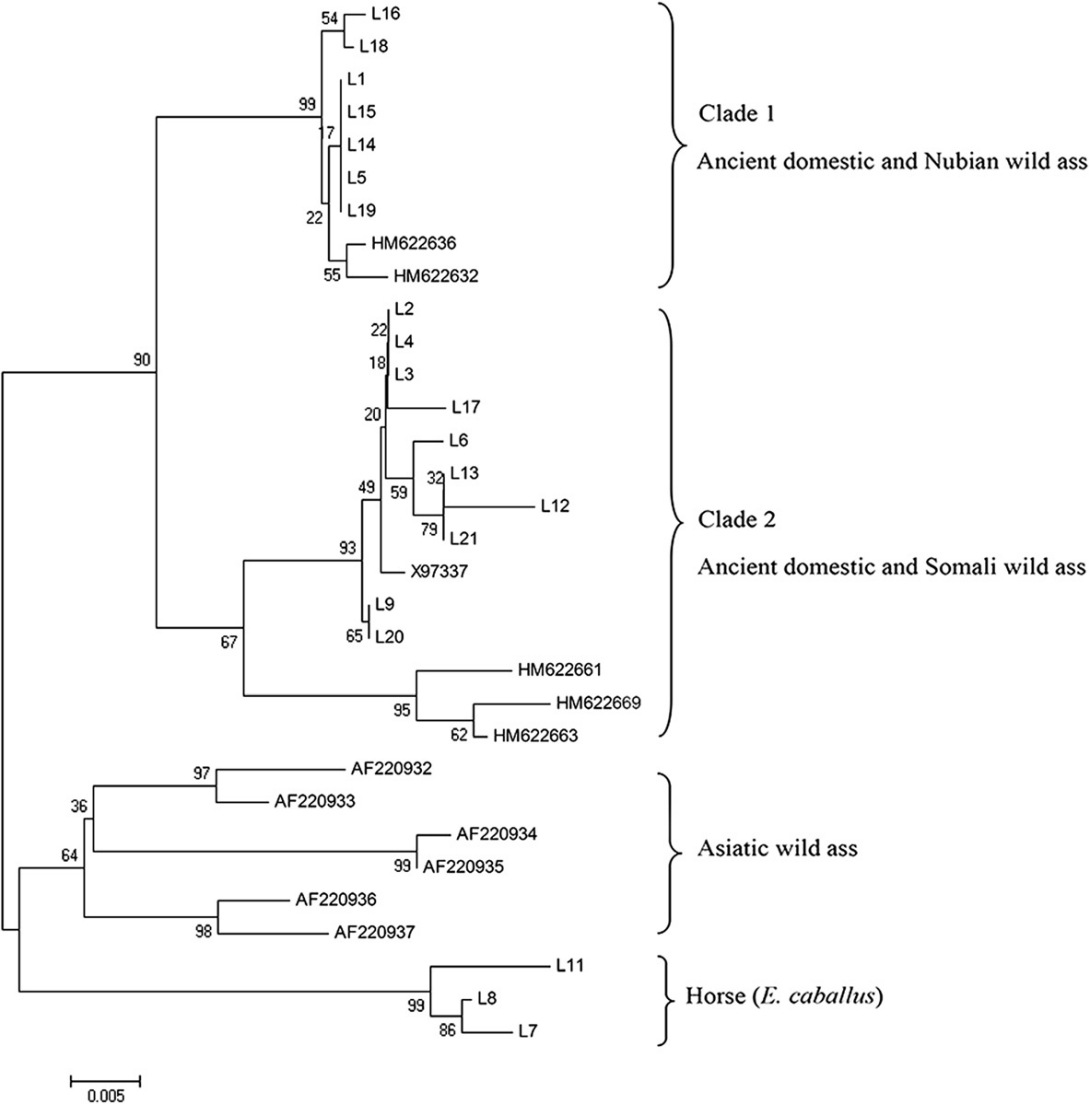
**Neighbor joining tree constructed from the mtDNA D-loop sequences of the ancient Chinese donkeys and horses.** The GenBank sequence X97337 was included as a reference sequence in the tree. Five African wild ass sequences were also added, including two Nubian wild asses (*E. a. africanus*) (HM622632 and HM622636), and three Somali wild asses (*E. a. somaliensis*) (HM622661, HM622663, and HM622669). The six Asiatic wild ass sequences were AF220932-AF220937, of which AF220932 and AF220933 belong to the species *E. kiang*, AF220934-AF220936 belong to the species *E. hemionus kulan*, and AF220937 belongs to the species *E. hemionus onager*.

The neighbor-joining tree was constructed using the 20 mtDNA D-loop sequences from the ancient samples (KM234980-KM234999), five African wild ass sequences (HM622661-622663, HM622636, and HM622669) [24], and six Asiatic wild ass sequences (AF220932-AF220937) [26]. It clearly shows that the domestic donkeys were divided into two distinct mtDNA haplogroups, Clade 1 and Clade 2 (Figure 1). Seven samples, including L1, L5, L14, L15, L16, L18, and L19, clustered in Clade 1, also called the Nubian lineage, while ten samples, including L2-L4, L6, L9, L12-L13, L17, and L20-L21, clustered in Clade 2, known as the Somali lineage. Three samples—L7, L8, and L11—were horses that grouped together as an outgroup to the donkeys in the phylogenetic tree. The results show that the African wild ass was the probable progenitor for ancient Chinese donkeys. These results support previous studies on the origins of the domestic donkey [6,9].

**Figure 2:**
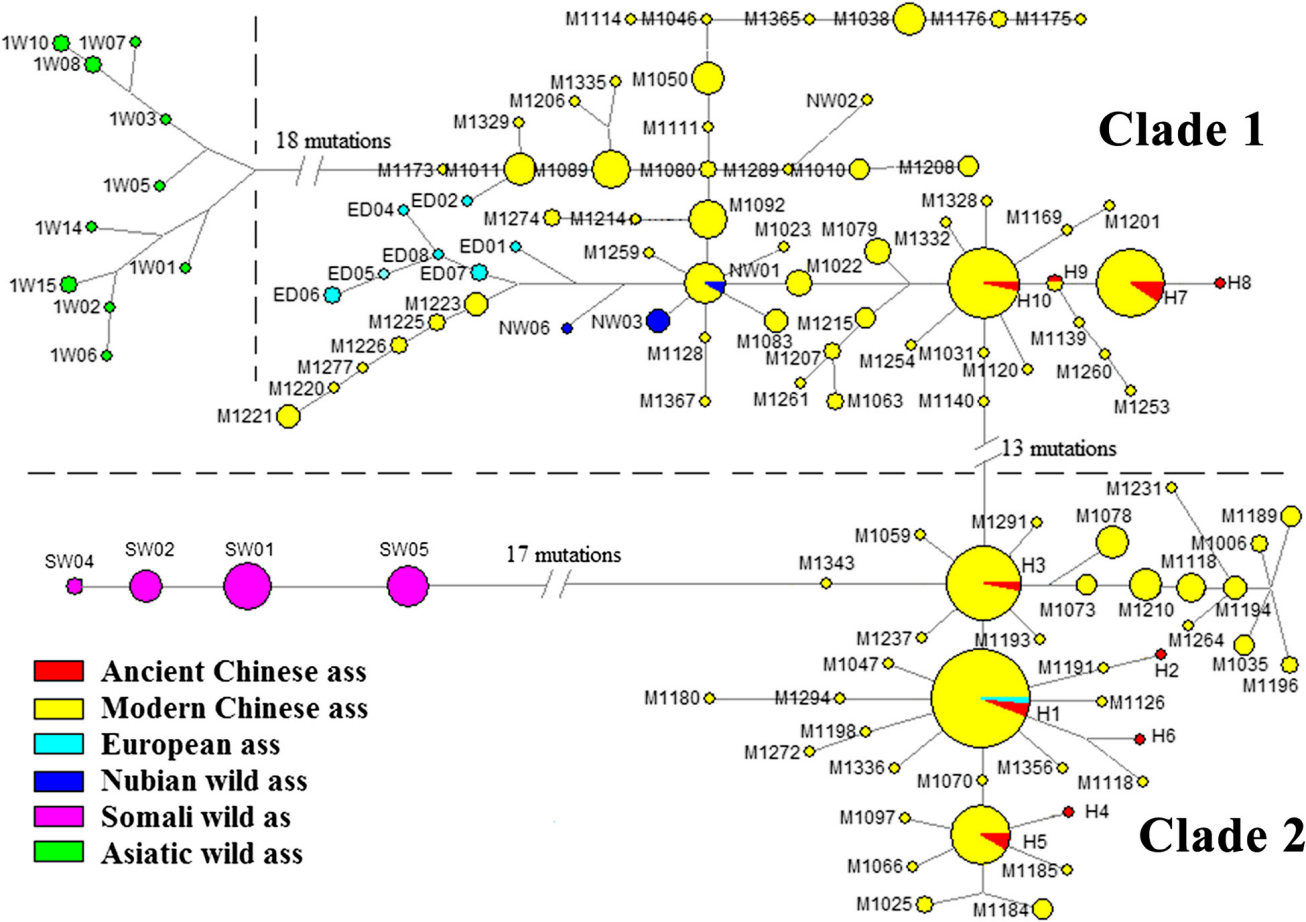
**Reduced median-joining network of 450 mtDNA D-loop sequences from domestic and wild asses.** The size of the circle is proportional to the haplotype frequency, and the branch length is proportional to the number of mutations.

Further analysis of the phylogenetic relationship between the 17 ancient donkeys involved comparing them to 433 previously published mtDNA D-loop sequences from donkeys around the world. These data were used to construct a reduced median network (Figure 2). The reduced median network revealed that there are two distinct lineages, as shown in the phylogenetic tree and the star-like phylogeny. Haplotype H1, which is found in four ancient donkeys, and haplotype H10, which is found in one ancient donkey, are located in the center of the Somali and Nubian lineages, respectively. The reduced median network shows that the Nubian wild ass clusters within the Nubian lineage, and the Somali wild ass clusters closest to the Somali lineage, but does not lie within the lineage and is considerably diverged from domestic donkeys. The results from this ancient DNA analysis support previous research that the Nubian wild ass is the wild ancestor of the maternal Nubian lineage [24]. While haplotypes H2, H4, H6, and H8 are unique, the other haplotypes found in ancient Chinese donkeys are also found in modern Chinese donkeys, which suggests that ancient Chinese donkeys contributed genetically to modern Chinese donkeys, at least in the maternal lineage.

## Discussion

### Maternal origin and dispersal route of Chinese domestic donkeys

In this study, the phylogenetic analysis of mtDNA D-loop sequences show that the ancient Chinese donkeys group into two clusters (Figures 1 and 2). One cluster with the Nubian wild ass is called the Nubian lineage and the other cluster closest to the Somali wild ass is called the Somali lineage, though the Somali wild ass is not within this lineage. Both of these lineages are distant from the Asiatic wild ass, which suggests that the maternal origin of Chinese donkeys is most likely from the African wild ass, but not the Asiatic wild ass. The results support the previous genetic research about the origin of the donkey, in which the donkey is the only ungulate domesticated solely in Africa about 5000 years ago [6].

This phylogenetic analysis including ancient Chinese donkeys resolves the issue about the maternal origin of Chinese domestic donkeys, but it also raises new questions. When were domestic donkeys introduced into China? What was the dispersal route of the ancient donkey into China? Do donkeys have any relationship with the Silk Road?

Historical records and DNA evidence from donkey remains provide some valuable information on the timing and dispersal route of donkeys into China. First, the archaeological evidence from Egypt suggests that domestic donkeys appeared in Africa about 5000 years earlier than the first known donkeys in China [6]. Second, Chinese historical records state that the donkey and mule were treasures rare in the northern and western regions of China 4000 years ago [27]. Third, the phylogenetic results for modern donkeys imply that the African wild ass is the probable ancestor of modern Chinese domestic donkeys [9,11,12]. Fourth, the ancient DNA analysis in this study supports that the ancient Chinese domestic donkeys are likely derived from the African wild ass. All of the above evidence implies that the donkey was domesticated from the African wild ass in Africa first. Then, the domestic donkey was introduced into northern and western China earlier than the Han dynasty (about 2,000 yr BP), before the Silk Road opened, perhaps in conjunction with ethnic migrations.

The number of ancient Chinese donkeys expanded gradually. First, Chinese historical records state that 4000 years ago, the donkey was a treasure that was rare and only found in the aristocratic and imperial palaces [7,20,27]. In this study, two ancient donkeys were found in Xi’an Wanke sites, which are dated to the Tang dynasty (1,160±30 yr BP). Seventeen suspected donkey remains were found in the Yanjialiang site of Inner Mongolia dated to the Yuan dynasty (800-600 yr BP). Historical records show that Xi’an was called Chang’ an in ancient times, and it was the Chinese capital as well as the Chinese political and economic center during the Tang Dynasty. The Silk Road is one of the most famous and important trade routes, and it began in the Han Dynasty and reached its golden age in the Tang Dynasty, facilitating trade between China and further west. It is worth noting that the two ancient samples were from the Xi’an Wanke site in Xi’an of China, which is dated to the Tang Dynasty. The combination of the historical record described above and the African origin of the ancient Chinese donkeys suggests that the increase of domestic donkeys was mainly to meet demand for the expansion of trade, and that domestic donkeys were used as commodities or for shipping goods during the Tang Dynasty along the Silk Road.

### Genetic relationship between ancient and modern Chinese domestic donkeys

It is worth noting that the mtDNA D-loop sequences of the Xi’an Wanke ancient samples, the Yanjialiang ancient samples, and the modern Chinese samples all had the same haplotypes, H3 and H7, which belonged to the Somali and Nubian lineages, respectively (Figure 2). In addition, the reduced medium network shows that haplotypes H1, H3, and H5 are located in the center of the Somali lineage, and haplotypes H7 and H10 are located in the center of the Nubian lineage. The ancient and modern Chinese donkeys all share these haplotypes, which suggests that the ancient Chinese donkeys contributed genetically to modern Chinese donkeys.

Phylogenetic analysis reveals that there is an abundance of genetic diversity in Chinese domestic donkeys. In this study, 17 ancient donkey samples contained ten different haplotypes. The haplotype diversity (Hd) and nucleotide diversity (π) values of the ancient Chinese donkeys were 0.919 and 0.01815, respectively, which suggests that there is abundant genetic diversity in the ancient Chinese donkey population. The Hd for ancient donkeys (0.919) is similar to that of the modern Inner Mongolian donkeys (0.9158) [28], which may be because most ancient Chinese donkeys are from the Yanjialiang site in Inner Mongolia.

There are different frequencies of each lineage in the ancient and modern populations. Of all ancient Chinese donkeys, seven samples were identified as the Somali lineage at 41.18%, and ten samples were identified as the Nubian lineage at 58.82%. At the Yanjialiang site, six of 14 samples belong to the Somali lineage (42.86%), while eight of 14 samples belong to the Nubian lineage (57.14%). In the Xi’an Wanke site, one sample belongs to the Somali lineage, while the other sample belongs to the Nubian lineage. In the Lantianxinjie site, the only sample L21 belongs to Nubian lineage. Thus, the Nubian lineage was slightly more predominant in ancient times. In contrast, the frequency of samples belonging to the Nubian lineage was slightly lower (43.85%) than that of the Somali lineage (56.15%) in modern donkeys [13]. Although ancient Chinese donkeys have contributed genetically to modern donkeys, there are still some differences in frequency for the maternal lineage.

## Conclusion

This study presents the first substantial analysis of genetic diversity in ancient Chinese donkeys using the mtDNA D-loop and the *cytb* gene, both of which provide important information about the maternal origin and domestication of Chinese donkeys. In conclusion, the maternal origin of the Chinese donkey is likely related to the African wild ass, and the domestic donkey was probably introduced into China earlier than the Han Dynasty (2,000 yr BP), before the Silk Road opened. The number of ancient Chinese donkeys was increased mainly to meet demand for the expansion of trade, and the donkeys were likely used as commodities or for shipping goods along the Silk Road during the Tang Dynasty, when the Silk Road reached its golden age. This study is the first to provide valuable ancient animal DNA evidence for ancient trade between African and Asian populations. The ancient DNA analysis of Chinese donkeys also sheds light on the dynamic process of maternal origins and dispersal in ancient Chinese donkeys.

## Methods

### Ethics statement

Ancient donkey samples were collected and sent to the ancient DNA laboratory of Jilin University for ancient DNA analysis under custody of the Research Center for Chinese Frontier Archaeology of Jilin University, Archaeological Research Institute of Shaanxi Province, and Institute for Archaeology and Relic Conservation of Xi’ an. They had the permission from the State Administration of Cultural Heritage who issued the permission for each excavation in China.

### Ancient material

**Figure 3:**
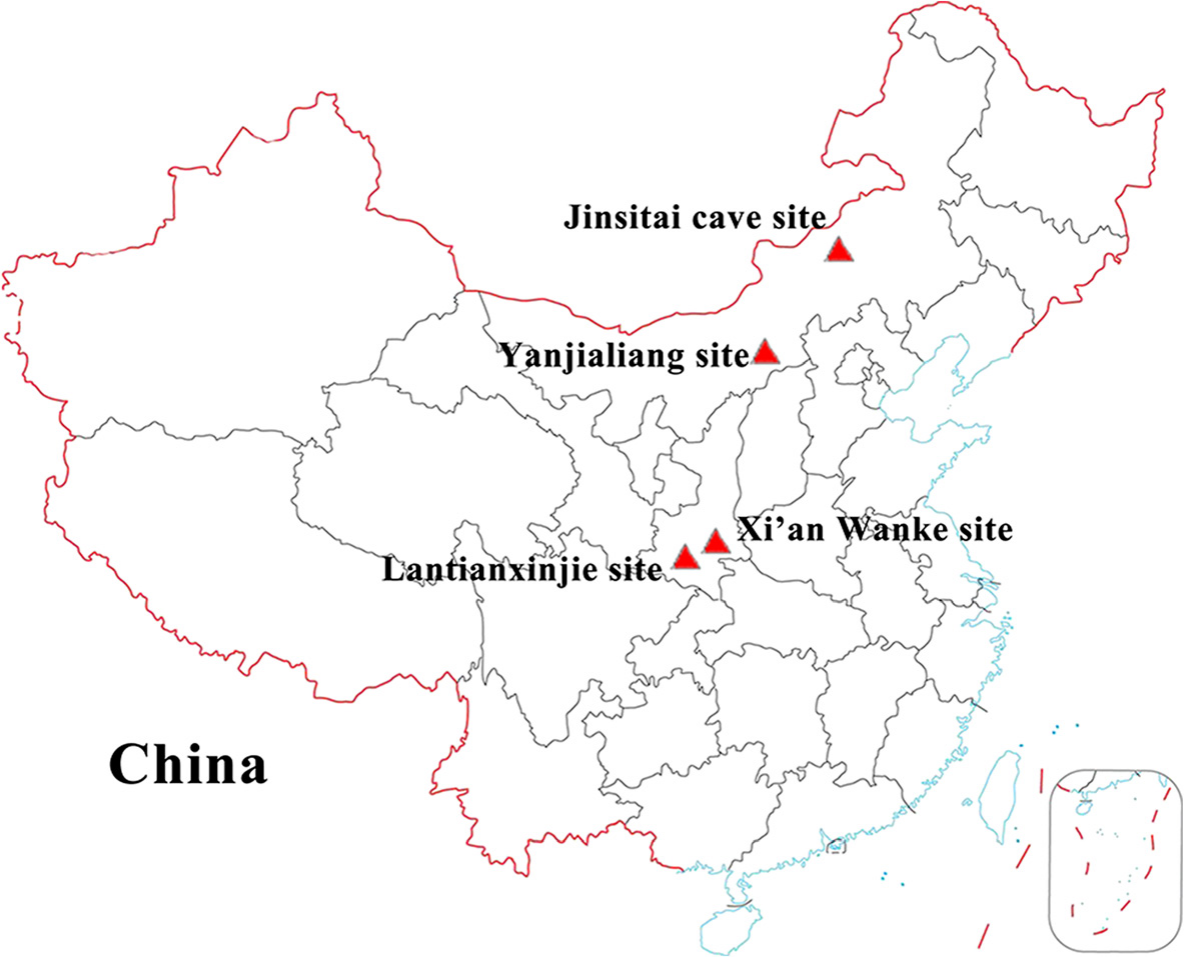
**Geographic location of the archaeological sites in this study.** Colored triangles refer to the archaeological site.

Twenty-one ancient samples were collected from four archaeological sites in China for this study (Table 1). Among these samples, 17 samples (L1- L9 and L11-L18) were from the Yanjialiang site in Inner Mongolia, which dates to 800-600 yr BP, during the Yuan dynasty [29]. Sample L10 was from the Jinsitai cave site in Inner Mongolia, which dates to 20,000-18,000 yr BP [30]. Sample L19 and L20 were from the Xi’an Wanke site in Shaanxi province, which dates to 1,160±30 yr BP using C14 dating, during the Tang dynasty. Sample L21 was from the Lantianxinjie site in Shaanxi province. The date of the sample was determined to be 568±50 yr BP using C14 dating. The geographic locations of the four archaeological sites in China where ancient donkey specimens were collected for sampling are shown in Figure 3.

### DNA extraction, PCR amplification and sequencing

Samples were handled using standard precautions for working with ancient DNA. Each sample was ground to powder with a freezer mill and incubated in 0.45 M EDTA (pH 8.0) and 0.25 mg/ml proteinase K overnight at room temperature under constant rotation. MtDNA was extracted from the bone or tooth of 21 ancient samples using the QIAquick® PCR Purification Kit.

Primers were designed to amplify overlapping segments ranging from 15424-16107 (684 bp) of the mtDNA D-loop and 14936-15383 (448 bp) of the *cytb* gene (Table 2). PCR was conducted on a 12.5 uL reaction with 2.5 mM MgCl_2_, 10 × PCR Buffer, 0.3 mM dNTPs, 1.0 g · L^−1^ BSA,0.75 umol · L^−1^ for each primer,1 Unit of Taq DNA Polymerase and 2.0 uL of DNA extraction. The PCR was performed on an Applied Biosystem (Verti™ 96-well Thermal Cycler), starting with pre-denaturation at 95°C for 5 min, followed by 32 cycles of 94°C (1 min), 50°C (30s), 72°C (1 min), and a final extension at 72°C (10 min). The concentration of Mg^2+^ and the annealing temperature were stringently adjusted in different PCRs of the 21 specimens. The Watson PCR Purification Kit (QIAamp®DNA Minikit) was used to purify the PCR products. Finally, the purified PCR products were sequenced via an ABI PRISM®310 Genetic Analyzer (Applied Biosystems, USA).

The contamination precautions in every step during the whole procedure were conducted in accordance with Cooper et al. [31]. The process of DNA extraction and PCR amplification was conducted in the ancient DNA laboratory at Jilin University, which is dedicated to ancient DNA studies. The sequencing and analytical work was conducted in the ancient DNA laboratory of the College of Life Science at Jilin University. To minimize environmental contamination, sterile gloves, mouth-mufflers, and laboratory coats were worn in all experiments and the external surfaces of specimens were removed by abrasion. To avoid cross-contamination, these tools were regularly changed and washed using steam sterilization. Furthermore, negative controls were used in each DNA extraction and PCR amplification.

### DNA analysis

To enlarge the sample size, 433 previous published sequences were downloaded from GenBank to construct the reduced median network: modern Chinese donkeys [AF531459-AF531470, AF532118-AF532126, DQ368497-DQ368596, AY666165-AY666169, DQ448878–DQ449023, and EF056034-EF056128]; European domestic donkeys [AF403063-AF403065, and AF416593-AF416599]; Asiatic wild ass [AF220932-AF220933, AF220934-AF220937, AY569539-AY569542, AY569551, GQ324612]; African Nubian wild ass [HM622627-HM622630, HM622632, HM622634, and HM622636]; and African Somali wild ass [HM622637-HM622669, HM622631, and AY569545-AY569547].

The sequences were aligned using Clustal X 1.83 [32]. The haplotype diversity (Hd) and nucleotide diversity (π) were estimated using DnaSP 5.10.01 [33]. The neighbor-joining tree was constructed using the Kimura-2 parameter model with 1000 bootstrapping replicates, so that the aligned sequences could be used to identify possible phylogenetic lineages in the MEGA 4.0 software [34]. A reduced median network was generated using NETWORK 4.6.1.2 [35,36].

### Availability of supporting data

The data set supporting the results of this article is available in the Dryad repository, doi:10.5061/dryad.c2v56 (http://datadryad.org/review?doi = doi:10.5061/dryad.c2v56). The mitochondrial DNA sequences have been submitted to GenBank and available under the accession numbers KM235000-KM235019 and KM234980- KM234999.
